# Physicochemical property changes of *Dendrobium officinale* leaf polysaccharide LDOP‐A and it promotes GLP‐1 secretion in NCI‐H716 cells by simulated saliva‐gastrointestinal digestion

**DOI:** 10.1002/fsn3.3341

**Published:** 2023-03-31

**Authors:** Jingfang Xiong, Jingyu Fang, Dongya Chen, Hong Xu

**Affiliations:** ^1^ Department of Geriatrics Zhejiang Hospital of Integrated Traditional Chinese and Western Medicine Hangzhou Zhejiang 310000 China; ^2^ Department of Food Science and Technology Zhejiang University of Technology Hangzhou Zhejiang 310000 China; ^3^ Department of Gastroenterology and Hepatology Zhejiang Hospital of Integrated Traditional Chinese and Western Medicine Hangzhou Zhejiang 310000 China

**Keywords:** *dendrobium Officinale* leaf, glucagon‐like peptide‐1, polysaccharide, simulated saliva‐gastrointestinal digestion, structure

## Abstract

A polysaccharide LDOP‐A with a molecular weight of 9.9 kDa was isolated and purified from *Dendrobium officinale* leaves by membrane separation, cellulose column, and dextran gel column. The Smith degradable products, methylation products, and nuclear magnetic resonance analysis showed that LDOP‐A may be composed of →4)‐Glc‐(1→, →3,6)‐Man‐(1→, and →6)‐Glc‐(1→sugar residues. In vitro, simulated digestion assays showed that LDOP‐A could be partially digested in the stomach and small intestine, and produced a large amount of acetic acid and butyric acid during colonic fermentation. Further cell experiment results illustrated that LDOP‐A‐I (LDOP‐A digested by gastrointestinal tract) could induce glucagon‐like peptide‐1 (GLP‐1) secretion in NCI‐H716 cells without showing any cytotoxicity.

## Graphical abstract

In order to inspect the anti‐diabetes effect of the polysaccharide extracted from *Dendrobium officinale* leaf named LDOP‐A, the present work first investigated the structure and simulated digestion of LDOP‐A in vitro and then explored secretion of GLP‐1 from endocrine L cells in vitro under the direct stimulation of digested LDOP‐A.

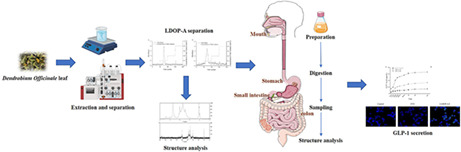

## 1 INTRODUCTION

Due to the increasing incidence of chronic diseases associated with excessive nutrition and unhealthy lifestyle, Chinese herbs have been widely investigated at home and abroad and show a beneficial effect for improving sub‐health status. *Dendrobium officinale* is an important edible, medicinal plant, enriched in polysaccharides, which are the main bioactive ingredients (Zhao et al., 2007). *Dendrobium officinale* leaf, the main tissue of whole plant, has the high efficacy of regulating immunity, anti‐oxidation, anti‐tumor, anti‐saccharification, and reducing blood sugar. *Dendrobium officinale* leaf owns a great potential medicinal value and may be used as raw material to create new drugs for the prevention and treatment of a variety of diseases.

Polysaccharides, a kind of natural macromolecular substance, is connected by a variety of monosaccharides through glycosidic bonds. It has many functional activities and high safety and is expected to become a natural active substance for the treatment of Type 2 diabetes (T2DM) (Chen et al., 2019). Pharmacological studies have proved that the activity of polysaccharides is related to the composition, molecular weight, residue type, and repeat unit of monosaccharides (Xie et al., 2020). As a natural active ingredient that is difficult to be digested and utilized, the structural changes and active structure of polysaccharides in gastrointestinal tract need further study (Koh et al., 2016). Short‐chain fatty acids (SCFA), a microbial fermentation product derived from dietary fiber, play an important medium in intestinal flora to affect host health. The available evidence suggests that polysaccharides can slow down or reverse the decrease of butyric‐acid‐producing bacteria and intestinal butyric acid levels in T2DM patients (Kumar et al., 2021; Qin et al., 2021). Some studies have shown that butyric acid can increase the secretion of Glucagon‐like peptide‐1 (GLP‐1) and peptide YY in the intestine, thus promoting the secretion of insulin in T2DM patients (Fan & Pedersen, 2020). GLP‐1 is a kind of incretin secreted under the stimulation of eating, which is released by L cells of the distal ileum and colon (Pan et al., 2021). Previous studies have indicated that plant polysaccharides have an excellent hypoglycemic effect by increasing the levels of insulin and GLP‐1 in streptozotocin‐induced diabetic rats (Lee et al., 2018). GLP‐1 can control the fluctuation of blood sugar by increasing insulin secretion, reducing glucagon level and appetite, and also can promote pancreatic islets β‐cell proliferation, differentiation, and reduction of β apoptosis (Kuang et al., 2020; Xie et al., 2020; Yang et al., 2021).

In order to detect the in vitro anti‐diabetes effect of the polysaccharide extracted from *Dendrobium officinale* leaf named LDOP‐A, the present study investigated the structure and simulated digestion of LDOP‐A in vitro and then explored secretion of GLP‐1 from endocrine L cells by direct stimulation of digested LDOP‐A.

## 2 MATERIALS AND METHODS

### 2.1 Extraction of *Dendrobium officinale* leaf polysaccharides

*Dendrobium officinale* leaves were purchased from Yueqing, Zhejiang Province. Eighty‐five percent ethanol was added into the dry leaf powder at the ratio of 1:10 and stirred by magnetic force for 8 h. The filter residue was collected. The decolorized powder was dissolved in distilled water at a dilution rate of 2:3 (v/v), extracted at 70°C for 2 h, repeated twice, combined with the filtrate, and centrifuged at 7140 *g* for 15 min. The supernatant was then concentrated and precipitated with 80% ethanol, standing at 4°C overnight, and centrifuged to obtain precipitation. Freeze dry the precipitate and dissolve it with distilled water under the condition of pressure of 0.2 MPa and flow rate of 30 L/h, and continuous ultrafiltration with 100 kDa membrane is carried out 5–10 times until the maximum permeation yield is reached. The filtrate was collected and freeze‐dried as described above.

#### 2.1.1 Purification of polysaccharides

Five‐hundred milligram of crude polysaccharides were dissolved in 20 mL distilled water and fractionated through a DEAE cellulose column (2.6 cm × 60 cm). Use distilled water, and 0.2 mol/L, 0.4 mol/L, and 0.8 mol/L NaCl solution to conduct gradient elution in turn. The elution rate is 1 mL/min. Collect 6 mL in each tube and 200 tubes in total. Dilute the eluent in the tube and measure the absorbance value at 490 nm according to the phenol–sulfuric acid method. Draw the elution curve with OD value as the ordinate, and combine the eluents of main peaks according to the curve. The collected DEAE samples were further purified by Sephadex G‐100 gel permeation column chromatography. Elute with distilled water at a flow rate of 0.5 mL/min, collect the eluent (5 mL/tube), determine the sugar content with phenol sulfuric acid method, and draw the elution curve. Collect the fraction containing main carbohydrate and concentrate and dry it to obtain LDOP‐A.

### 2.2 Structural identification of polysaccharides

#### 2.2.1 Analysis of Smith degradation products of LDOP‐A

Fifteen milligram LDOP‐A was dissolved in 8 mL 30 M sodium periodate solution, then allowed reaction under 4°C without light. Samples were taken every 12 h and measured the absorbance at UV 233 nm. After the OD value is stable, 0.4 mL ethylene glycol is added to the system to stop the reaction. The reaction solution was dialysised with distilled water for 24 h, and then concentrate it to 2–3 mL. 100 mg NaBD_4_ was added into the concentrated solution, and placed at room temperature for 12 h, then neutralized with glacial acetic acid. Twice the volume of methanol was added to the solution and dried at 45°C under reduced pressure. TFA (3 mL, 2M) was added into the reduced product and then hydrolyzed at 110°C for 5 h, evaporate it with methanol. Four milliliter acetic anhydride–pyridine (1:1) was mixed into the hydrolyzate, filled with nitrogen, and then placed at 110°C for 1 h for acetylation reaction. After the reaction, the mixture was extract 3 times with n‐hexane, the extraction liquid was combined and evaporated to dryness under reduced pressure, the residue was dissolving with water and passed 0.22 μm membrane filtration to conduct GC–MS analysis. The chromatographic column is HP‐5MS (30 m × 0.25 mm × 0.25 μm), with high‐purity helium as carrier gas, and the flow rate is controlled to 1 mL/min. The sample injection volume is 0.2 μ 50. The injection port temperature is set to 210°C. The heating procedure is as follows: the initial temperature is 140°C and maintained for 6 min; first, raise the temperature to 190°C at the rate of 2°C/min; then, the temperature was raised to 210°C at the rate of 1°C/min and maintained for 5 min.

#### 2.2.2 Analysis of methylation products of LDOP‐A

Fifteen milligram LDOP‐A was completely dissolved in 3 mL of DMSO. NaOH powder (100 mg) was added into the reaction system; after reaction at 18–20°C for 30 min, the reaction is ended by freezing in ice bath. Then, 2 mL methyl iodide was slowly added to the mixture under nitrogen atmosphere, and stand for 16 h to ensure complete methylation, repeat 2–3 times. The reaction solution was washed repeatedly with 2 mL chloroform for four times and the organic phase were combined and dried with anhydrous sodium sulfate. TFA (2 mL, 2M) was added to the residue. After reacting at 120°C for 2 h, two times the volume of methanol was added to the mixture and steam three times. 2–3 mL distilled water was added into the evaporated system, the pH was adjusted between 10 and 12 with 0.1 mol/L NaOH solution, then 100 mg NaBD_4_, was added and reacted for 2 h, the glacial acetic acid was used to neutralize excess NaBD_4_ until there are no bubbles. The hydrolyzate was acetylated and analyzed by GC–MS. Column and analysis procedure are the same as above.

#### 2.2.3 ^1^H‐NMR and ^13^C‐NMR analysis of LDOP‐A

Thirty milligram LDOP‐A was dissolved in 0.5 mL of D_2_O to conduct the structural identifications by ^1^H‐NMR and ^13^C‐NMR analysis and obtain one‐dimensional NMR spectrum.

### 2.3 Simulated digestion and in vitro fermentation of polysaccharides

#### 2.3.1 Simulated digestion

The in vitro simulated saliva‐gastrointestinal digestion of LDOP‐A was performed based on previous methods (Wu, Nie, et al., 2021; Wu, Yuan, et al., 2021). Briefly, Saliva samples (age range 20–25 years) were provided by two volunteers who had not received antibiotic treatment within 3 months. Volunteers were asked not eat or drink at morning until saliva was collected. LDOP‐A solution (8 mg/mL) was mixed with 100.0 mL simulated saliva at equal volume and oscillated at a rate of 100 rpm in water bath oscillator at 37°C. After 0.25, 0.5, and 1.0 h of incubation, 5.0 mL digested solution was collected and boiled to inactivate the enzyme and investigate the content of reducing sugar using 3,5‐dinitrosalicylic acid method. In addition, a part of mixed liquid after 1.0 h was centrifuged, precipitated with ethanol, redissolved, and dialyzed to obtain LDOP‐A‐S.

After salivary digestion, 50 mL of simulated gastric solution (112.5 mg gastric lipase and 106.2 mg pepsin added to 450 mL gastric electrolyte solution) was added to 50.0 mL mixture after saliva digestion at the ratio of 1:1 (v/v) (Jagadeesan et al., 2022), and the pH was immediately adjusted to pH 3.0 using 0.1 M HCl. After 0.5, 1.0, 2.0, 4.0, and 6.0 h of incubation at 37°C, 5 mL digested solution was again determined, and LDOP‐A‐G was obtained.

Add 200.0 g bile salt solution (4%, w/w), 100.0 g trypsin solution (7%, w/w), and 13.0 mg trypsin into 100.0 g intestinal electrolyte solution, and fully mix them to obtain a simulated small intestine solution (Jagadeesan et al., 2022). The pH of the mixture after saliva‐gastric digestion was adjusted to pH 7.0 by 0.1 M NaOH, and the simulated small intestine solution was added at a ratio of 3: 10 (v/v). After 0.5, 1.0, 2.0, 4.0, and 6.0 h of incubation at 37°C, 5 mL digested solution was again determined, and LDOP‐A‐I was obtained.

#### 2.3.2 Determination of reducing sugar content and SCFAs content

LDOP‐A‐I was further used for fermentation. The mixed fecal samples of healthy C57 mice were collected and diluted to 10% (w/v) with normal saline solution, and the diluent is filtered with sterile cotton cloth. 1.0 mL of filtrate was add to a 9.0 mL of fermentation medium containing 1.0 mg LDOP‐A‐I, and the mixture was cultured in a constant temperature shaking flask at 37°C. Blank control group was designed; 1.0 mL of filtrate was added into 9.0 mL fermentation medium. The fermentation samples were collected at 0 h and 48 h, respectively, and the content of SCFAs was analyzed by gas chromatography. The temperature rise procedure of GC is as follows: the temperature rises from 60°C to 100°C at the rate of 5°C/min, and then rises to 145°C at the rate of 10°C/min (Dou et al., 2019).

#### 2.3.3 Determination of structural changes of polysaccharides before and after simulated digestion

The monosaccharide composition of LDOP‐A was determined by high‐performance liquid chromatography (HPLC) (Xie et al., 2022). One‐hundred milligram of dried sample was dissolved in 5 mL of trifluoroacetic acid (TFA) and then hydrolyzed at 120°C for 2 h. After cooling the solution to room temperature, the reaction mixture was purged with N_2_, and then dissolved in 4 mL ultrapure water. 400 μL methanol solution containing 1‐phenyl‐3‐methyl‐5‐pyrazolone (0.5 mol/L, 3‐methyl‐1‐phenyl‐2‐pyrazolin‐5‐one, PMP) and 400 μL NaOH solution (0.3 mol/L) were added to 1 mL hydrolyzed solution, then put in 70°C water bath for 2 h, and neutralized with 500 μL HCl solution (0.3 mol/L) and washed with chloroform three times as after treatment. The supernatant was collected and filtered with 0.22 μm microporous filter membrane. C_18_ column (5 μm, 4.6 mm × 250 mm, Thermo Fisher, USA) was used for analysis and detected at 245 nm with UV detector. The HPLC conditions are as follows: the column temperature is 30°C; mobile phase A is 0.1 mol/L phosphate buffer (pH = 6.4), mobile phase B is acetonitrile, and the flow rate is 1.0 mL/min. The gradient elution procedure is set as follows: 0–34.5 min, 82% A; 34.5–45 min, 20–50% A; 45–51 min, 50% A; 51–53 min, 50–82% A; and 53–58 min, 82% A.

Cold field‐emission scanning electron microscope was used at 2 kV accelerating voltage and the sample surface was observed at ×2000 image magnification.

### 2.4 Cell experiment

#### 2.4.1 Cell culture

NCI‐H716 cell was obtained from ATCC. The cell growth medium was RPMI 1640 medium, in which 10% FBS and 1% penicillin/streptomycin were added.

#### 2.4.2 Cell viability assay

Cytotoxicity was assessed by Cell Counting Kit‐8 (CCK‐8) assay. The operation is the same as reported (Pan et al., 2021). The number of multiple holes in each group is set to 5. Incubate at 37°C for 24 h, wash with preheated PBS three times, and add 100 μL of culture medium containing 0.5 mg/mL CCK‐8 into each well. After incubated at 37°C for 24 h and washed with preheated PBS three times, the cells were incubated with 100 μL of culture medium containing 0.5 mg/mL CCK‐8 at 37°C in the dark for 2 h. Read the absorbance at 490 nm on the microplate reader.

#### 2.4.3 GLP‐1 secretion assay

The secretion level of GLP‐1 was detected after the NCI‐H716 cells were stimulated by LDOP‐A‐I in vitro. LDOP‐A‐I, sodium acetate, and sodium butyrate were dissolved in PBS. NCI‐H716 cells with 1 × 10^6^ cells/mL densities were treated and inoculated into 96‐well plates coated with Matrix, and different concentrations of LDOP‐A‐I (0, 25, 100, 200, and 500 μg/mL) were added, respectively, sodium acetate (0, 2, 4, and 8 mmol/L) and sodium butyrate (0, 250, 500, and 1000 μmol/L) in a humidified incubator at 5% carbon dioxide and 37°C for 24 h (Pan et al., 2021). Add 50 μg/mL benzylsulfonyl fluoride to the supernatant and centrifuge at the speed of 1000 *g* for 15 min, and collect the supernatant. The level of GLP‐1 in the supernatant was determined by human glucagon‐like peptide quantitative detection kit (ELISA). The standard curve of absorbance and GLP‐1 content are calculated as: *y* = 121.2*x*–18.596, *R* ^2^ = 0.9709.

Set the NCI‐H716 with a density of 1 × 10^6^ cells/mL cultured in 48‐well culture plate coated with Matrix for 48 h, and then stimulated with LDOP‐A‐I (100 or 200 μg/mL). The content of GLP‐1 in cell supernatant was determined by the method above after adding polysaccharides for 0 min, 15 min, 30 min, 60 min, 90 min, 2 h, 8 h, and 16 h.

#### 2.4.4 Binding of LDOP‐A‐I to NCI‐H716 cells

The FITC‐conjugated f‐LDOP‐A‐I was prepared by labeling LDOP‐A‐I with fluorescein isothiocyanate at the reduction end. NCI‐H716 cells were spread on the glass cover slide with a density of 1 × 10^4^ cells/mL. Cells were stained for 1 h with culture medium, free FITC (20 μg/mL), and f‐LDOP‐A‐I (200 μg/mL) at 4°C. Then, use PBS diluted 4,6‐diamino‐2‐phenylindole (DAPI) to 1:1000 for nuclear restaining. After standing at room temperature for 15 min, observe and photograph with fluorescence scanning microscope (Olympus, Tokyo, Japan).

## 3 RESULTS AND DISCUSSION

### 3.1 Structural characterization of LDOP‐A

**FIGURE 1:**
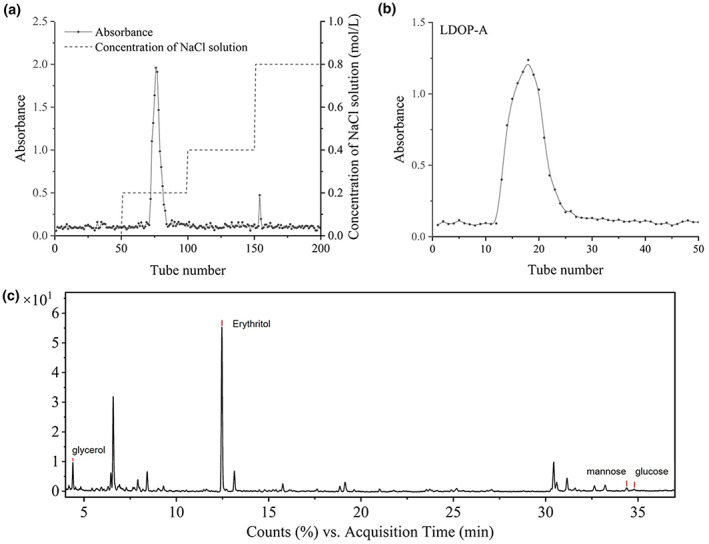
Elution curves of LDOP‐A by DEAE column (a) and Sephadex G column (b); GC–MS chromatograms of Smith degradable products of LDOP‐A (c).

After separation and purification by DEAE and Sephadex gel column, homogenous polysaccharide LDOP‐A was obtained, and the structures were characterized. LDOP‐A is composed of glucose, mannose, galactose, and arabinose with a molar ratio of 5.3:3.5:1.0:0.2. The content of polysaccharide determined by phenol–sulfuric acid method is 90.29%, and the ultraviolet full‐scan spectrum shows that it does not contain nucleic acid and protein. It can be seen from the separation of DEAE in Figure 1a that the LDOP‐A is slightly polar, which is related to the content of uronic acid, but the content of acidic polysaccharide in its structures is not high. It can be seen from the separation diagram of dextran gel (Figure 1b) that the peak shape of the separated polysaccharide is symmetrical, and it is a polysaccharide with uniform molecular weight.

Periodic acid can selectively oxidize the hydroxides in polysaccharide molecules to produce formic acid and corresponding aldehydes. After the oxidation products of periodate are successively reduced and acid hydrolyzed (Smith degradation), the connection position and type of glycosidic bond can be inferred according to the peak situation. The Smith degradation products of polysaccharides of LDOP‐A were analyzed by GC–MS (Figure 1c). The results showed that in addition to ethylene glycol, a large amount of erythritol and a small amount of glycerol were detected in the degradation products of LDOP‐A, and the ratio was 7.96:1. According to the production of glycerol and erythritol, it can be inferred that LDOP‐A is mainly 1→4 or 1→4,6 bond type, and there are a few 1→, 1→2, 1→6, or 1→2,6 bond types.

**FIGURE 2:**
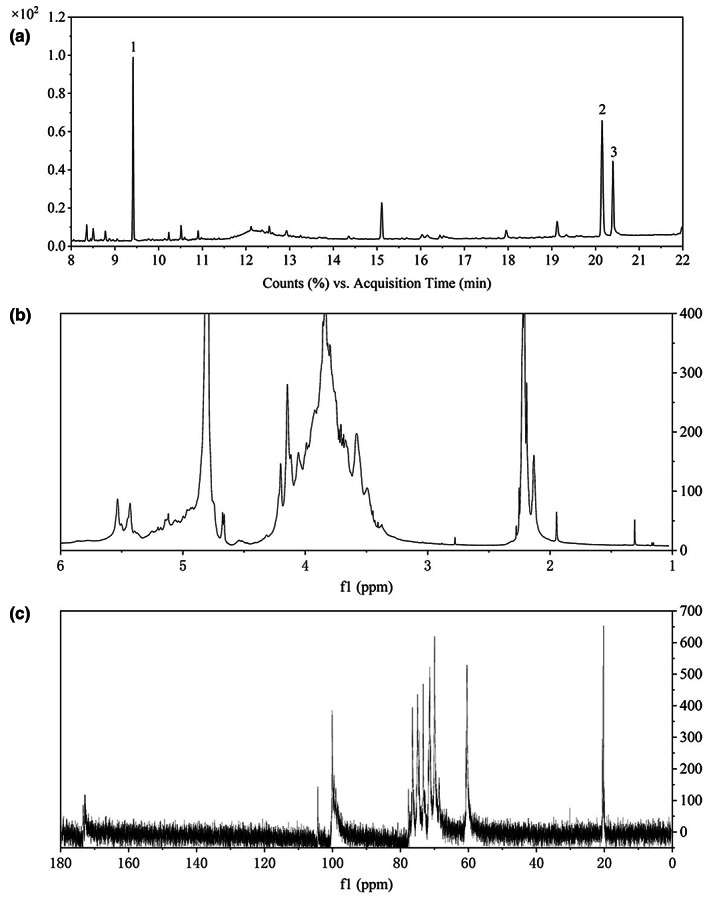
GC–MS chromatograms of methylation product of LDOP‐A (a), 1H NMR (b), and 13C NMR (c) spectrum of LDOP‐A.

Methylation is an important means to determine the position of glycosidic bonds between monosaccharides in polysaccharides. The determination results of GC–MS are shown in Figure 2. According to the fragment information of corresponding retention time and the comparison with the CCRT database, the type of monosaccharide residues can be inferred. In LDOP‐A, sugar residues →4)‐Glc‐(1→, →3,6)‐Man‐(1→ and →6)‐Glc‐(1→ are the main types of sugar residues, accounting for 43.99%, 31.46%, and 24.54% of the total glycosidic bonds in turn, which was basically consistent with monosaccharide composition. In addition, there are also some signals of branching structures, but the link position and detailed characteristics of branched chains need further experimental confirmation.

The ^1^H NMR and ^13^C NMR spectra of LDOP‐A are shown in Figure 2b, c. Four signals were found at δ 5.43, 5.33, 4.68, and 4.57 ppm in the downfield region (Figure 2b). In the ^13^C NMR spectrum of LDOP‐A (Figure 2c), anomeric carbon signals were found at δ 104.34, 100.10, 99.55, and 99.36 ppm in the downfield region. The existence of acetyl group in LDOP‐A can also be omitted from the NMR spectra, the signal at around δ 20.55 ppm was methyl carbon, while the chemical shift at δ 173.33 ppm indicated the presence of ‐O‐acetyl [9].

### 3.2 Simulated digestion of LDOP‐A in vitro

**FIGURE 3:**
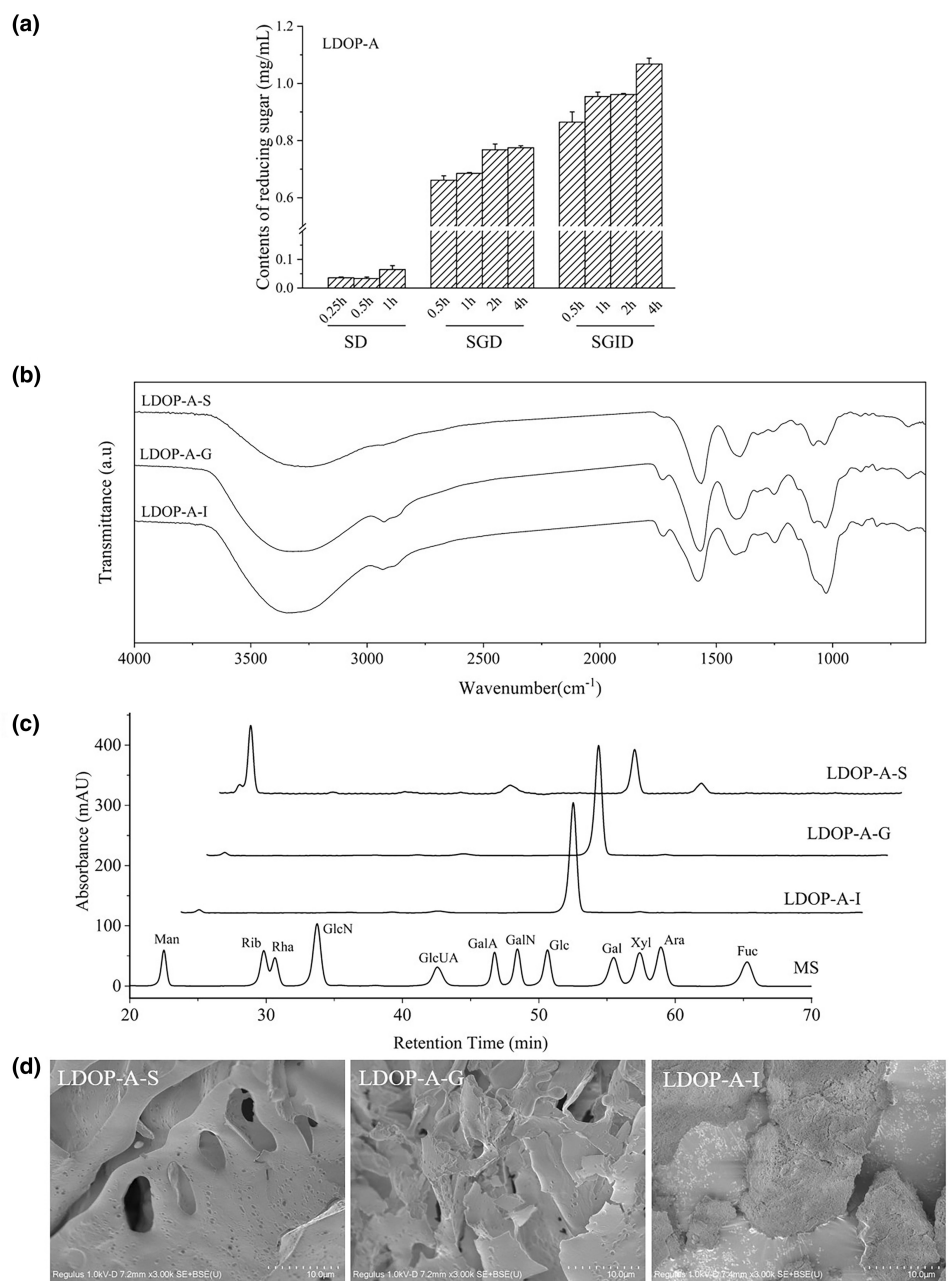
Reducing sugar content (a), infrared spectrum (b), monosaccharide composition (c), and surface structure (d) during digestion of LDOP‐A.

In the process of polysaccharide digestion in vivo, the breaking of glycosidic bonds will lead to more reduction ends exposed as a whole, which will increase the content of reducing sugar (Kasipandi et al., 2019). The main function of saliva is to partially digest the starch in food. As shown in Figure 3a, in the simulated saliva digestion process, the content of reducing sugar has not increased significantly and a significant increase of CR was observed after gastrointestinal digestion, which indicates that LDOP‐A was difficult to be hydrolyzed in saliva. Gastric juice and intestinal juice are the main digestive juice of human body. After 4 h of gastric digestion, the content of reducing sugar in LDOP‐A increases to 0.77 mg/mL, and sharply increased to 1.07 mg/mL after 4 h of intestinal digestion, which indicates that LDOP‐A may be partially degraded during gastrointestinal digestion. This may be related to the extremely low pH value of gastric juice (pH = 2.0) and the hydrolysis of polysaccharides by more digestive enzymes in small intestinal juice.

The structural features of LDOP‐A‐S, LDOP‐A‐G, and LDOP‐A‐I were evaluated by the FT‐IR (Figure 3b). The broad peak at 3360 cm^−1^ is caused by the stretching vibration of a large number of O–H in the polysaccharide, and the simultaneous absorption peaks near 809 cm^−1^ and 871 cm^−1^ are the unique absorption peaks of glucomannan. After gastric digestion, obvious absorption peaks at 2918 cm^−1^ and 1727 cm^−1^ appeared, corresponding to the stretching vibration of C = O in free carboxyl (− COOH) and esterified carboxyl (− COOR). And the peak intensity of LDOP‐A‐G at 1033 cm^−1^ and 1081 cm^−1^ also increased significantly in LDOP‐A‐G and LDOP‐A‐I, which indicates that the structure of the polysaccharide of LDP‐A has changed greatly after gastric digestion and intestinal juice digestion.

Generally speaking, the glycosidic bond on the branch chain of the segment is easier to break, while polysaccharide with larger molecular weight is more difficult to be absorbed and utilized in the digestive tract, which may be related to the relatively regular and orderly structure and its low solubility and easy formation of gel (Payling et al., 2020). During in vitro digestion, the monosaccharide composition of LDOP‐A after oral digestion is almost no different from that of untreated polysaccharides, which is consistent with the change in reducing sugar content. However, after simulated gastric digestion, the monosaccharide composition of LDOP‐A changed significantly (Figure 3c). The monosaccharide composition of LDOP‐A‐G and LDOP‐A‐I was determined as glucose, and there were also trace amounts of mannose and glucuronic acid. Compared with undigested LDOP‐A, the mannose peak basically disappeared after gastric digestion, which may be because mannose on the polysaccharide branch chain can be hydrolyzed at low pH (Wu, Nie, et al., 2021; Wu, Yuan, et al., 2021). Combined with the results of the glycosidic bond composition of LDOP‐A, we can predict that after digestion in the stomach and small intestine, LDOP‐A‐I will become a glycan composed of →4)‐Glc‐(1→ and →6)‐Glc‐(1→sugar residues.

It can be seen from the scanning electron microscope in Figure 3d that a large number of pore structures can still be seen in the LDOP‐A‐S after saliva digestion, while the pore structure of polysaccharide in LDOP‐A‐G basically disappears and becomes fine flakes (Figure 3d). After simulating small intestine digestion, the polysaccharide is further degraded, and small pores appear on the surface of LDOP‐A‐I. This may be related to the branched‐chain structure of LDOP‐A is decomposed by gastric juice, which is consistent with the disappearance of mannose in the monosaccharide composition of LDOP‐A‐G.

### 3.3 In vitro fermentation of polysaccharides

On basis of simulated digestion in vitro, the effect of indigestible polysaccharide LDOP‐A‐I on SCFAs production of intestinal microorganisms was further explored. In general, non‐digestible polysaccharides enter the intestine and are decomposed and metabolized as a carbon source for the microbiota in the intestine. The derived end products from anaerobic fermentation of microorganisms include gases and SCFAs, mainly acetate, propionate, and butyrate, and their proportions have many beneficial effects in several different tissues (Koh et al., 2016). Therefore, SCFAs can reflect the relationship between the decomposition of polysaccharides and the structure of intestinal microbiota.

More and more evidence has shown that the occurrence of diabetes is related to the insufficient production of butyrate in the intestine because butyrate may play a key role in maintaining the integrity of intestinal epithelium and the metabolic balance of human body and intestinal microorganisms (Kumar et al., 2021). As shown in Table 1, acetic acid is the most abundant organic acid produced by microorganisms after the metabolism of *Dendrobium officinale* leaf polysaccharide, and the rest are propionic acid, butyric acid, and n‐valeric acid. After 48 hours of fermentation, the level of total SCFAs in each group increased significantly. The total SCFAs in the blank control group and LDOP‐A‐I were 15.59 and 26.76 mmol/L, respectively. Among them, the content of butyric acid produced by LDOP‐A‐I was significantly higher after 48 hours of fermentation of up to 1.55 mol/L. These results indicated that LDOP‐A can significantly increase the level of total SCFA during simulated fermentation in vitro, especially the levels of acetic acid and butyric acid. The increase in butyric acid level can reduce the occurrence of diabetes or the intestinal imbalance of diabetic patients (Jia et al., 2017). However, whether the LDOP‐A from *Dendrobium officinale* leaf can significantly regulate the flora in vivo and the production of SCFA needs further research.

**TABLE 1:** Concentration of short‐chain fatty acids.

Group	Time	SCFAs (mmol/L)						
		Acetic acid	Propionic acid	Butyrate acid	Isobutyric acid	Valeric acid	Isovaleric acid	Total SCFA
Blank control	0	ND	ND	ND	ND	ND	ND	ND
	24	12.80 ± 0.87^a^	2.41 ± 0.79^a^	0.08 ± 0.03^a^	0.10 ± 0.09^a^	0.18 ± 0.04^a^	1.18 ± 0.078^b^	15.59 ± 1.12^a^
LDOP‐A‐I	0	ND	ND	ND	ND	ND	ND	ND
	48	19.16 ± 3.02^b^	3.97 ± 2.17^b^	1.55 ± 1.33^c^	0.69 ± 0.37^c^	0.36 ± 0.27^b^	0.02 ± 0.001^a^	26.76 ± 3.58^c^

*Note*: Different lowercase letter (a‐c) in the same line indicate significant difference (*p* < .05).

### 3.4 Effects of LDOP‐A‐I on GLP‐1 secretion in NCI‐H716 cells

The results of GLP‐1 secretion showed that a certain amount of sodium acetate (4–8 mmol/L) and sodium butyrate (0.5‐1 mmol/L) could significantly stimulate the secretion of GLP‐1 in NCI‐H716 cells. It can be inferred that LDOP‐A can act as carbon source to change the dominant strains of intestinal flora and stimulate the secretion of GLP‐1 by increasing the production of acetic acid and butyric acid.

**FIGURE 4:**
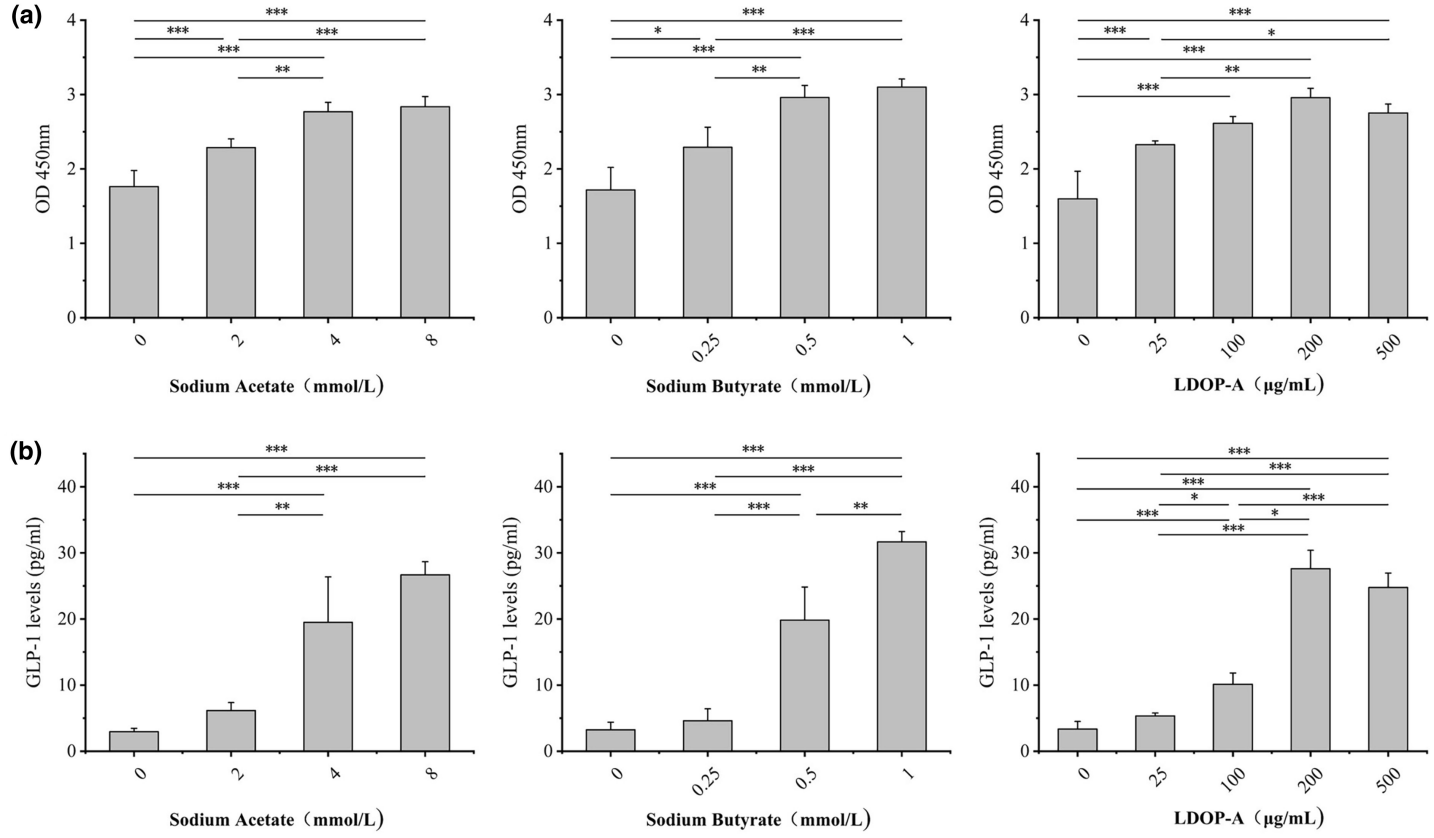
Cytotoxicity on NCI‐H716 cells (a) and GLP‐1 secretion of NCI‐H716 cells (b).

Recent studies have shown that some natural polysaccharides can promote GLP‐1 secretion through direct interaction with intestinal epithelial cells (Pan et al., 2021). In the present study, NCI‐H716 cells were exposed to LDOP‐A‐I to confirm the direct effects of digested LDOP‐A on GLP‐1‐secreting L cells. The obtained results indicated that LDOP‐A‐I at low concentrations (25 μg/mL) can obviously promote the proliferation of NCI‐H716 cells. 200–500 μg/mL LDOP‐A‐I can also significantly promote the proliferation of NCI‐H716 cells without obvious toxicity (Figure 4a). Simultaneously, LDOP‐A‐I cannot significantly increase the level of GLP‐1 at low concentrations, but LDOP‐A at high concentrations (100, 200 or 500 μg/mL) can significantly increase the secretion of GLP‐1, and the secretion of GLP‐1 in different concentrations are 3.02, 8.27, and 7.41 times of the control group, respectively. These results support the fact that LDOP‐A‐I may directly stimulate GLP‐1 secretion in intestinal L cells. It can be inferred that LDOP‐A can have an effect on the increase of insulin after meals, reducing glucagon level, or reducing appetite.

### 3.5 LDOP‐A‐induced GLP‐1 secretion

In order to further verify the direct stimulatory effect of polysaccharides on cells, the GLP‐1 secretion level of cells within 16 h was detected in this experiment. The secretion process of GLP‐1 showed that LDOP‐A could rapidly stimulate the secretion of GLP‐1 between 15 min and 2 h, and then maintain a high level. At 2 h, the secretion level of GLP‐1 in 200 μL/mL LDOP‐A group can reach 31.16 ± 1.26 pg/mL.

**FIGURE 5:**
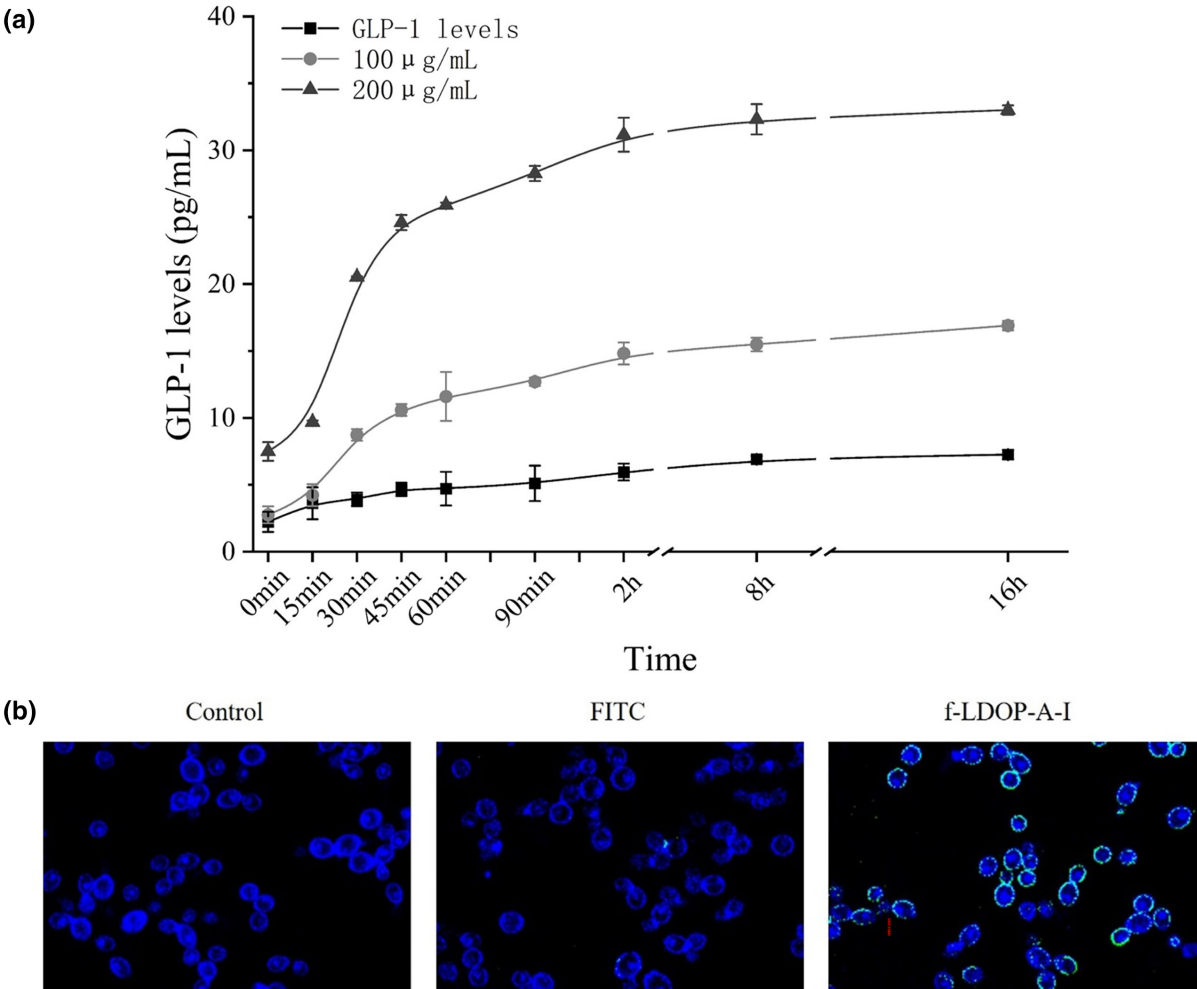
LDOP‐A (a) promotes the secretion of GLP‐1 by NCI‐H716 cells; binding analysis of LDOP‐A and NCI‐H716 cells (b).

It is generally believed that natural polysaccharides express their biological activity by being recognized by cell receptors (Wang et al., 2017). Figure 5b shows the binding mode of LDOP‐A‐I and cell NCI‐H716. The results showed that after NCI‐H716 cells were incubated with f‐LDOP‐A‐I for 1 h, a large number of f‐LDOP‐A appeared on the surface of the cells, while the fluorescence intensity around the cells stained with free FITC was relatively weak. Therefore, it can be inferred that NCI‐H716 cells have receptors responsible for recognizing LDOP‐A on the surface. The literature shows that the sweetener receptor is composed of two different G‐protein‐coupled receptors T1R2 and T1R3, which are widely distributed in the gastrointestinal tract outside the mouth and play an important role in regulating the secretion of GLP‐1 (Xie et al., 2019, 2020). Therefore, it can be speculated that LDOP‐A after gastrointestinal digestion can stimulate NCI‐H716 cells to secrete GLP‐1 through surface stimulation, but whether its receptors are T1R2 and T1R3 still needs further experimental proof.

## 4 CONCLUSION

The activity of natural polysaccharides is related to its structure. Polysaccharides from plants are usually administered orally, and the digestive tract (especially the intestine) has been proven to be an important part of polysaccharides regulating body functions. Polysaccharide was the main component in *Dendrobium Officinale* leaf, the contents were measured as 8.99–16.55%, respectively (Zhou et al., 2014), while the total flavonoid contents in *D. officinale* leaves collected only ranged from 7.66 to 9.50 mg/g dried weight (Zhong et al., 2022). Therefore, understanding the digestive characteristics of polysaccharides is conducive to the in‐depth study of their functions. As a natural macromolecular substance, plant polysaccharides are mainly fermented and utilized by intestinal flora. SCFAs, as metabolites of flora, are important mediators for intestinal flora to affect host health (Ulven, 2012). The remission of T2D by polysaccharides may be through a variety of ways, one of which is to stimulate the secretion of GLP‐1 by intestinal L cells. Therefore, the present study explored the structural changes in LDOP‐A after oral administration and its effect on promoting SCFAs production and GLP‐1 secretion, thus providing new evidence for LDOP‐A as anti‐T2DM medicated diet for patients.

In this preliminary work, we presented a comprehensive understanding of the structural change process of LDOP‐A after oral administration and explored the GLP‐1 secretion‐promoting effect of digested polysaccharides, which will provide a theoretical basis for the clinical application of LDOP‐A. It has been proved that after oral administration, LDOP‐A is partially digested in the stomach and small intestine, the mannose fragments are absorbed and recycled into the blood, and the indigestible part can produce a large amount of acetic acid and butyric acid through intestinal fermentation. Moreover, polysaccharides in the gut can promote the secretion of GLP‐1 by NCI‐H716 cells. However, the interaction between polysaccharides and NCI‐H716 cells still needs further systematic study. In the future, the digestion of polysaccharides will be explored with animal experiments in vivo. In addition, whether the degradation products (smaller fragments) of LDOP‐A or the flora secondary metabolites change induced by LDOP‐A  plays a more important role in ameliorating T2DM is also our future research direction.

## AUTHOR CONTRIBUTIONS

**Jingfang Xiong:** Conceptualization (equal); data curation (equal); formal analysis (equal); investigation (equal); methodology (equal); visualization (equal); writing – original draft (equal). **Jingyu Fang:** Data curation (equal); investigation (equal); methodology (equal); resources (equal); software (equal); visualization (equal). **Dongya Chen:** Data curation (equal); software (equal); visualization (equal). **Xu Hong:** Conceptualization (equal); funding acquisition (equal); methodology (equal); project administration (equal); supervision (equal); validation (equal); writing – review and editing (equal).

## ACKNOWLEDGEMENTS

The authors are highly thankful to Zhejiang Hospital of Integrated Traditional Chinese and Western Medicine for the financial support. Authors are also thankful to the Department of Food Science and Technology, Zhejiang, China, for providing the laboratory facility in conducting a part of the experimental work of this study.

## FUNDING INFORMATION

This work was supported by the Hangzhou Science and Technology Bureau (20201203B175) and the Chinese Medicine Science and Technology Project of Zhejiang Province (2023ZL558).

## CONFLICT OF INTEREST STATEMENT

The authors declare no conflict of interest.

## INFORMED CONSENT

Informed consent was obtained from all subjects involved in the study.

## Data Availability

Data are contained within the article.

## References

[fsn33341-bib-1023] Chen, Y. , Liu, D. , Wang, D. , Lai, S. , Zhong, R. , Liu, Y. , Yang, C. , Liu, B. , Sarker, M. R. , & Zhao, C. (2019). Hypoglycemic activity and gut microbiota regulation of a novel polysaccharide from Grifola frondosa in type 2 diabetic mice. Food and Chemical Toxicology, 126, 295–302. 10.1016/j.fct.2019.02.034 30826407

[fsn33341-bib-0001] Dou, Z. , Chen, C. , & Fu, X. (2019). Digestive property and bioactivity of blackberry polysaccharides with different molecular weights. Journal of Agricultural and Food Chemistry, 67, 12428–12440. 10.1021/acs.jafc.9b03505 31668067

[fsn33341-bib-0002] Fan, Y. , & Pedersen, O. (2020). Gut microbiota in human metabolic health and disease. Nature Reviews Microbiology., 19, 55–71. 10.1038/s41579-020-0433-9 32887946

[fsn33341-bib-0003] Jagadeesan, G. , Muniyandi, K. , Manoharan, A. L. , Nataraj, G. , & Thangaraj, P. (2022). Understanding the bioaccessibility, alpha‐amylase and alpha‐glucosidase enzyme inhibition kinetics of *Allmania nodiflora* (L.) r.Br. Ex Wight polyphenols during *in vitro* simulated digestion. Food Chemistry, 372, 131294. 10.1016/j.foodchem.2021.131294 34638068

[fsn33341-bib-0004] Jia, L. , Li, D. , Feng, N. , Shamoon, M. , Sun, Z. , Ding, L. , Zhang, H. , Chen, W. , Sun, J. , & Chen, Y. Q. (2017). Anti‐diabetic effects of clostridium butyricum CGMCC0313.1 through promoting the growth of gut butyrate‐producing bacteria in type 2 diabetic mice. Scientific Reports, 7, 7046. 28765642 10.1038/s41598-017-07335-0 PMC5539151

[fsn33341-bib-0005] Kasipandi, M. , Manikandan, A. , Sreeja, P. S. , Suman, T. , Saikumar, S. , Dhivya, S. , & Parimelazhagan, T. (2019). Effects of *in vitro* simulated gastrointestinal digestion on the antioxidant, α‐glucosidase and α‐amylase inhibitory activities of water‐soluble polysaccharides from Opilia amentacea roxb fruit. LWT, 111, 774–781. 10.1016/j.lwt.2019.05.079

[fsn33341-bib-0006] Koh, A. , De Vadder, F. , Kovatcheva‐Datchary, P. , & Backhed, F. (2016). From dietary fiber to host physiology: Short‐chain fatty acids as key bacterial metabolites. Cell, 165, 1332–1345. 10.1016/j.cell.2016.05.041 27259147

[fsn33341-bib-0007] Kuang, M. T. , Li, J. Y. , Yang, X. B. , Yang, L. , Xu, J. Y. , Yan, S. , Lv, Y. F. , Ren, F. C. , Hu, J. M. , & Zhou, J. (2020). Structural characterization and hypoglycemic effect via stimulating glucagon‐like peptide‐1 secretion of two polysaccharides from *Dendrobium officinale* . Carbohydrate Polymers, 241, 116326. 10.1016/j.carbpol.2020.116326 32507202

[fsn33341-bib-0008] Kumar, V. , Khare, P. , Devi, K. , Kaur, J. , Kumar, V. , Kiran Kondepudi, K. , Chopra, K. , & Bishnoi, M. (2021). Short‐chain fatty acids increase intracellular calcium levels and enhance gut hormone release from STC‐1 cells via transient receptor potential Ankyrin1. Fundamental & Clinical Pharmacology, 35, 1004–1017. 10.1111/fcp.12663 33636045

[fsn33341-bib-0009] Lee, Y. , Lee, C. , Choung, J. , Jung, H. , & Jun, H. (2018). Glucagon‐like peptide 1 increases beta‐cell regeneration by promoting alpha‐ to beta‐cell transdifferentiation. Diabetes, 67, 2601–2614. 30257975 10.2337/db18-0155

[fsn33341-bib-0010] Pan, L. , Xu, M. , Wang, Q. , Zou, X. , Han, Y. , & Zhou, Z. (2021). Long‐term drench of exopolysaccharide from *Leuconostoc pseudomesenteroides XG5* protects against type 1 diabetes of NOD mice via stimulating GLP‐1 secretion. Journal of the Science of Food and Agriculture, 102, 2023–2031. 10.1002/jsfa.11541 34558071

[fsn33341-bib-0011] Payling, L. , Fraser, K. , Loveday, S. M. , Sims, I. , Roy, N. , & McNabb, W. (2020). The effects of carbohydrate structure on the composition and functionality of the human gut microbiota. Trends in Food Science & Technology, 97, 233–248.

[fsn33341-bib-0012] Qin, W. , Ying, W. , Hamaker, B. , & Zhang, G. (2021). Slow digestion‐oriented dietary strategy to sustain the secretion of GLP‐1 for improved glucose homeostasis. Comprehensive Reviews in Food Science and Food Safety, 20, 5173–5196. 10.1111/1541-4337.12808 34350681

[fsn33341-bib-0013] Ulven, T. (2012). Short‐chain free fatty acid receptors FFA2/GPR43 and FFA3/GPR41 as new potential therapeutic targets. Frontiers in Endocrinology, 3, 111. 10.3389/fendo.2012.00111 23060857 PMC3462324

[fsn33341-bib-0014] Wang, K. , Cheng, F. , Pan, X. , Zhou, T. , Liu, X. , Zheng, Z. , Luo, L. , & Zhang, Y. (2017). Investigation of the transport and absorption of Angelica sinensis polysaccharide through gastrointestinal tract both *in vitro* and in vivo. Drug Delivery, 24, 1360–1371. 10.1080/10717544.2017.1375576 28920748 PMC8240978

[fsn33341-bib-0016] Wu, D.‐T. , Nie, X.‐R. , Gan, R.‐Y. , Guo, H. , Fu, Y. , Yuan, Q. , Zhang, Q. , & Qin, W. (2021). *In vitro* digestion and fecal fermentation behaviors of a pectic polysaccharide from okra (*Abelmoschus esculentus*) and its impacts on human gut microbiota. Food Hydrocolloids, 114, 106577. 10.1016/j.foodhyd.2020.106577

[fsn33341-bib-0017] Wu, D. T. , Yuan, Q. , Guo, H. , Fu, Y. , Li, F. , Wang, S. P. , & Gan, R. Y. (2021). Dynamic changes of structural characteristics of snow chrysanthemum polysaccharides during *in vitro* digestion and fecal fermentation and related impacts on gut microbiota. Food Research International, 141, 109888. 10.1016/j.foodres.2020.109888 33641944

[fsn33341-bib-0018] Xie, H. , Fang, J. , Farag, M. A. , Li, Z. , Sun, P. , & Shao, P. (2022). *Dendrobium officinale* leaf polysaccharides regulation of immune response and gut microbiota composition in cyclophosphamide‐treated mice. Food Chem X, 13, 100235. 10.1016/j.fochx.2022.100235 35499019 PMC9039934

[fsn33341-bib-0019] Xie, S. Z. , Shang, Z. Z. , Li, Q. M. , Zha, X. Q. , Pan, L. H. , & Luo, J. P. (2019). *Dendrobium huoshanense* polysaccharide regulates intestinal lamina propria immune response by stimulation of intestinal epithelial cells via toll‐like receptor 4. Carbohydrate Polymers, 222, 115028. 10.1016/j.carbpol.2019.115028 31320099

[fsn33341-bib-0020] Xie, S. Z. , Yang, G. , Jiang, X. M. , Qin, D. Y. , Li, Q. M. , Zha, X. Q. , Pan, L. H. , Jin, C. S. , & Luo, J. P. (2020). Polygonatum cyrtonema Hua polysaccharide promotes GLP‐1 secretion from enteroendocrine L‐cells through sweet taste receptor‐mediated cAMP signaling. Journal of Agricultural and Food Chemistry, 68, 6864–6872. 10.1021/acs.jafc.0c02058 32456438

[fsn33341-bib-0021] Yang, Z.‐M. , Wang, Y. , & Chen, S.‐Y. (2021). Astragalus polysaccharide alleviates type 2 diabetic rats by reversing the glucose transporters and sweet taste receptors/GLP‐1/GLP‐1 receptor signaling pathways in the intestine‐pancreatic axis. Journal of Functional Foods, 76, 104310. 10.1016/j.jff.2020.104310

[fsn33341-bib-0022] Zhao, Y. , Son, Y. O. , Kim, S. S. , Jang, Y. S. , & Lee, J. C. (2007). Antioxidant and anti‐hyperglycemic activity of polysaccharide isolated from *dendrobium chrysotoxum Lindl* . Journal of Biochemistry Molecular Biology, 40, 670–677. 17927899 10.5483/bmbrep.2007.40.5.670

[fsn33341-bib-0023] Zhong, C. , Tian, W. , Chen, H. , Yang, Y. , Xu, Y. , Chen, Y. , Chen, P. , Zhu, S. , Li, P. , & du, B. (2022). Structural characterization and immunoregulatory activity of polysaccharides from *Dendrobium officinale* leaves. Journal of Food Biochemistry, 46, e14023. 10.1111/jfbc.14023 34873736

[fsn33341-bib-0024] Zhou, G. F. , Pang, M. X. , Chen, S. H. , Lv, G. Y. , & Yan, M. Q. (2014). Comparison on polysaccharide content and PMP‐HPLC fingerprints of polysaccharide in stems and leaves of *Dendrobium officinale* . China Journal of Chinese Materia Medica, 39, 795–802. 25204167

